# Nuclear Import of Hepatitis B Virus Capsids and Genome

**DOI:** 10.3390/v9010021

**Published:** 2017-01-21

**Authors:** Lara Gallucci, Michael Kann

**Affiliations:** 1 Laboratoire de Microbiologie Fondamentale et Pathogénicité, University of Bordeaux, UMR 5234, F-33076 Bordeaux, France; lara.gallucci@u-bordeaux.fr; 2 CNRS, Microbiologie Fondamentale et Pathogénicité, UMR 5234, F-33076 Bordeaux, France; 3 Centre Hospitalier Universitaire de Bordeaux, Service de Virologie, F-33076 Bordeaux, France

**Keywords:** nuclear transport, genome release, hepatitis B virus, nuclear pore, nucleoporin

## Abstract

Hepatitis B virus (HBV) is an enveloped pararetrovirus with a DNA genome, which is found in an up to 36 nm-measuring capsid. Replication of the genome occurs via an RNA intermediate, which is synthesized in the nucleus. The virus must have thus ways of transporting its DNA genome into this compartment. This review summarizes the data on hepatitis B virus genome transport and correlates the finding to those from other viruses.

## 1. Introduction

All retroviruses and practically all DNA viruses (exception *Poxviridae* family) need the nuclear machinery of the host cell for their replication. As a pararetrovirus, hepatitis B virus (HBV) requires multiple nuclear enzymatic activities, which are only partially understood but late activities needed for mRNA synthesis are evident. Also obvious are all enzymes that are involved in the repair of the relaxed circular virion DNA (rcDNA) to the covalently closed circular DNA (cccDNA), which is the template for transcription. Up to now, a few host factors in rcDNA-to-cccDNA conversion were identified: tyrosyl-DNA-phosphodiesterase 2 (TDP2), which releases the viral polymerase from the rcDNA [1] and the DNA polymerase κ (POLK) but also polymerase λ (POLL) and η (POLH), which are involved in repair of the single stranded part of the rc genome [2].

Amongst the viral mRNAs is the RNA pregenome, which is the template for translation of the viral capsid protein (core protein, Cp) and the viral polymerase (Pol). Pol binds to a specific structure on the pregenome called ε and the Pol-RNA complex becomes encapsidated by the assembling Cp, forming an immature, RNA-containing capsid. This initiates reverse transcription and subsequent second strand DNA synthesis [3]. Mature capsids but not RNA-containing capsids are enveloped by the viral surface proteins [4]. Infections with a duck hepatitis B virus mutant unable to code for a surface protein led to increased cccDNA copy number from 50 to 150 per cell [5,6] indicating that the progeny rcDNA capsids transport the genome to/into the nucleus as the virion-derived capsids during initial infection. For HBV the situation is however, less clear as the cccDNA copy number is much smaller. Considering that there is no proof for nuclear entry of rcDNA from progeny capsids, it cannot be concluded that cytoplasmic capsids derived from infection are identical with those being newly synthesized.

**Figure 1:**
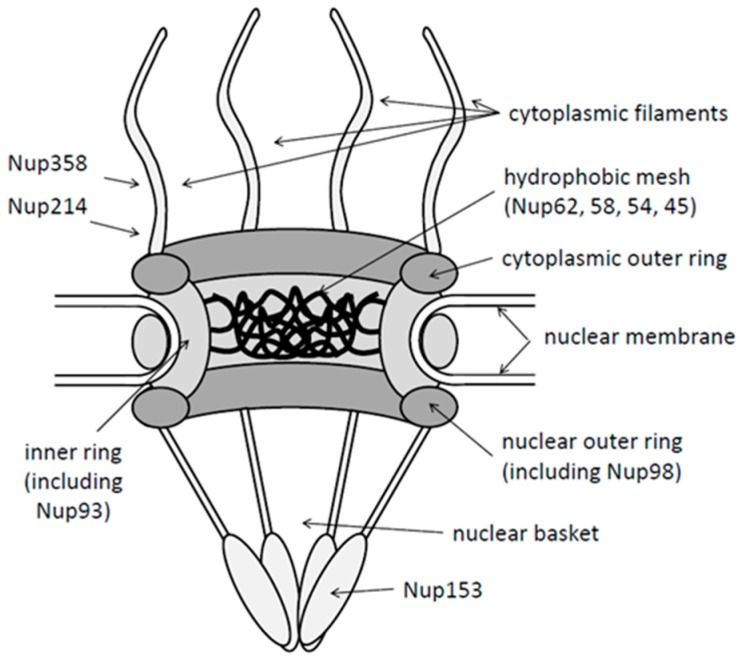
Schematic presentation of the nuclear pore complex (NPC) with key structures. Cytoplasm: top, nucleus: bottom of the figure. Nup: nucleoporins.

With a life span of approximately 200 days, hepatocytes are rarely dividing. Nuclear entry of the genome by passive trapping, as it was described for the human T-lymphotropicvirus-1 (HTLV-1) [7] and human papillomaviruses [8], would be thus inefficient. In contrast, HBV has to highjack cellular transport pathways as cellular transport factors facilitating nuclear import via the nuclear pore complexes (NPCs; see Figure 1). Throughout all viruses, one alternative pathway was described for the simian virus 40 (SV40), which cannot only pass via nuclear pores [9,10,11,12,13] but potentially disrupts the inner nuclear membrane after being internalized into the ER lumen [14].

## 2. Nuclear Pores and Nuclear Import Receptors

### 2.1. Structure of Nuclear Pores

NPCs are macromolecular complexes of >125 MDa having an octagonal rotation symmetry. NPCs are phylogenetically well conserved even between distant species like humans and *Xenopus leavis*. NPCs are composed of ~30 different proteins collectively called nucleoporins (Nups) (reviewed in [15]). Some Nups are organized in a symmetric ring in the nuclear envelope forming the pore. Other Nups exhibit an asymmetric distribution as e.g., Nup358 and Nup214, which are part of eight fibers emanating from the cytoplasmic face of the pore, and Nup153 and Tpr, which make up the so-called nuclear basket (reviewed in [16]). Nups are not stably incorporated into NPCs but vary substantially in their NPC residence times, which ranges between 13 s and 70 h [17,18]. Nups are not only essential for nuclear import and export but also for differentiation during embryogenesis, cell cycle, and intranuclear chromatin distribution [19,20]. One third of the Nups contains phenylalanine-glycine (FG)-repeats, which are essential for cell viability and which form a diffusion barrier inside the pore [21,22,23]. Passive diffusion through the NPC is limited to small molecules of up to 5 nm [24], while active transport of macromolecules, with a few exceptions, depends on nuclear transport receptors, collectively called importins or exportins (karyopherins). Active transport allows the translocation of ~1000 macromolecules per second and NPC [25] and the number of NPCs per nucleus varies upon the metabolic activity of the cell [26] (400 in Purkinje cells and 18,500 in oligodrendrocytes [27]). The NPC copy number is further regulated by the cell cycle, being higher during G2 phase than G1 phase (e.g., 8.5 to 5 NPCs/µm^2^ nuclear envelope in HeLa cells [28]). In electron microscopy, the channel forming the nuclear pore has a diameter of 40 nm and the maximal cargo size is 39 nm [29], which restricts import and export of large structures as viral capsids.

### 2.2. Nuclear Import Receptors

Nine nuclear import receptors (also called karyopherins; Kaps) which exhibit different cargo-specificities, have been described so far (reviewed in [30]). Most import receptors bind directly to their cargo as exemplified by transportin-1 (Kapβ2), which interacts with a proline-tyrosine (PY) nuclear localization signal (NLS). This signal_,_ has been first characterized in the C-terminal M9 domain of the heterogeneous nuclear ribonucleoprotein A1 (hnRNP A1) leading to the name M9 signal [31,32]. In contrast, importin β (Kapβ1) interacts either directly with cargos comprising an importin-β-binding domain (IBB) or it binds indirectly to classical NLSs via the adapter protein importin α (Kapα). Importin α comprises an IBB for importin β-interaction and a site for the interaction with classical NLSs. While a canonical IBB comprises 13 basic amino acids in seven clusters distributed over 39 residues [33,34], classical NLSs are formed by clusters of typically (four to) six basic amino acids, which can be mono- or bipartite. The NLS prototype is found on SV40 T-antigen (SV40TAg [35,36]) exhibiting the amino acid sequence PKKKRKV [35]. A certain selectivity of nuclear import is achieved by the diversity of importin α isoforms, which show preferences for some NLSs. There are seven importin α subfamilies, which are all expressed in adult tissues except importin α6, which is testis-specific (reviewed in [37]).

## 3. Nuclear Import of Macromolecules

### 3.1. Nuclear Import Using Import Receptors

Classical active nuclear import is initiated by the interaction of the import receptor with the NLS on the cargo. This interaction can be regulated by different mechanisms: post-translational modifications can directly affect import receptor cargo-binding. This was shown for the phosphorylation of threonine 124 on the SV40TAg, which is directly adjacent to the NLS and which inhibits the nuclear import [38]. Another form of inactivation is the intermolecular masking as exemplified by the NLS of nuclear factor κB (NFκB), which is hidden by binding of IκB and which becomes accessible after IκB degradation [39]. Intramolecular NLS masking was described for nuclear factor of activated T-cells (NF-ATc) in which the NLS is hidden by association with phosphorserines [40].

Interaction with the cargo changes the structure of the import receptor [41,42], allowing interaction with Nups. First of the Nups is Nup358 (also known as RanBP2), which localizes in the extremity of cytoplasmic filaments extruding the NPCs. The subsequent step of translocation through the nuclear pore, which is filled by a hydrophobic mesh, is not fully elucidated. A number of mechanisms have been proposed, all involving FG-repeats of Nups in the central channel (Nups 98, 93, 62, 58, 54 and 45) and which interact with transport receptors. The “polymer brush model”, for instance, suggests that movements of the unfolded FG-Nups sweep away macromolecules [43,44,45]; the so called “collapse model” suggests a collapse of FG-repeats [46,47], and the “hydrophobic gel model”, also called “saturated model”, postulates that transport factors bind to the FG-repeats dissolving the FG-repeat cross-links [21,48].

Nuclear import is terminated in the nuclear basket where the cargo-import receptor-complex binds to Nup153 for then being dissociated by interaction between the import receptor and the ras-related nuclear protein (Ran) in its GTP-bound form. While the cargo diffuses deeper into the nucleus, the import receptor-RanGTP-complex is exported through the NPC. On the cytoplasmic face of the NPC, GTP is hydrolyzed to GDP, which dissociates RanGDP from the import receptor, resulting in recycling of the import receptor. Recycling of Ran requires then nuclear import of RanGDP using the nuclear transport factor 2 (NTF2) [49], which is followed by the replacement of GDP by GTP. This exchange reaction is catalyzed by the chromatin-bound Ran guanine nucleotide exchange factor (RanGEF; also termed regulator of chromosome condensation, RCC1). The driving force of the import reactions is the RanGTP concentration, which is 1000 fold higher in nucleus than in the cytoplasm [50].

### 3.2. Import Receptor-Independent Pathways

There are a few reports describing alternative pathways as the one for the calcium-binding proteins calmodulin and calreticulin [51]. Translocation is mediated by direct interaction with NPC components and is independent of carrier molecules. Nevertheless, a coexistence with an import receptor-dependent pathway is assumed. Moreover, calmodulin and calreticulin themselves can act as nuclear import receptors, as they import the transcription factors SRY and SOX9 [52]. Calreticulin can also act as a nuclear export factor, which was exemplified for the glucocorticoid receptor, the thyroid hormone receptor α1 and some viral proteins as e.g., HTLV-1 protein Tax [53,54,55,56].

## 4. Capsid Disassembly and Import of Other Viral Genomes

### 4.1. Capsids Larger than the Maximal Transport Diameter of the NPC

The release of viral genomes with a nuclear replication step largely depends on the surrounding structure, which can have the form of a capsid. As viral genomes comprise pathogen-associated molecular patterns (PAMPs) they will be sensed by cytosolic or membrane-bound pattern recognition receptors (PRRs) [57]. It must be thus assumed that the later the genome is released on its way to the nucleus, the lower the risk of triggering innate immunity is. Thus, most DNA- and retroviruses ensure the transport of their genome within a closed protein shell until their arrival at the nuclear envelope. This is well established for adenoviruses (Ads) and herpes viruses such as the human herpes simplex virus-1 (HSV-1). Moreover, there is growing evidence that also human immunodeficiency virus (HIV) capsids, which are thought to liberate the preintegration complex (PIC) of DNA and attached viral proteins from the capsid in the cytosol, binds to the NPC prior to PIC release [58].

**Figure 2:**
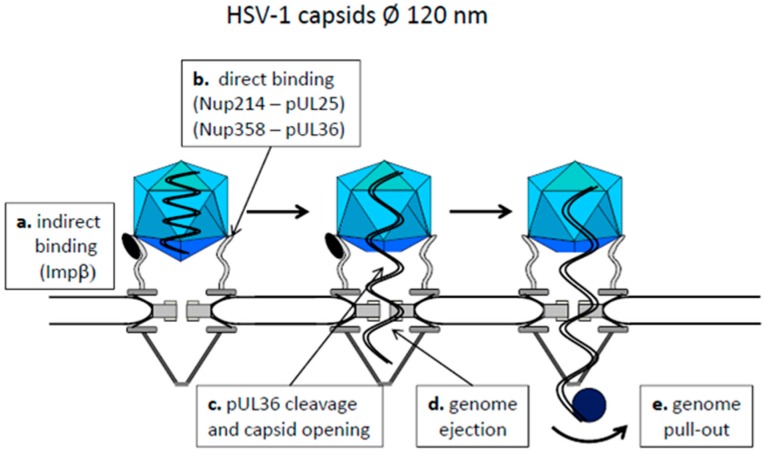
Model of capsid attachment to the NPC and nuclear genome release of herpesviruses. Cytoplasm: top, karyoplasm: bottom. The capsids are shown as blue icosahedra, the genome as a double waved line in black. Importin β (Impβ): black ellipse. The cellular RNA polymerase, transcribing the immediate early genes is depicted as blue sphere.

Herpes viruses enter the cell by fusion of the viral membrane with the plasma- or endosomal membrane (reviewed in [59]). This leads to release of the capsid, which stays attached to several tegument proteins [60,61]. Ojala et al., showed that the docking of the capsids to NPCs is mediated by importin β [62] (Figure 2a). Other reports describe a direct HSV capsid NPC-binding via the inner tegument protein pUL25 interacting with the cytoplasmic filament protein Nup214 [63] or by pUL36Nup358-interaction [64,65] (Figure 2b). Herpesviral capsids, being 120 nm in diameter, cannot pass the pore intact but become opened at a site opposed to the nuclear pore (Figure 2c) by a mechanism that remains enigmatic. Using permeabilized cells Ojala et al. showed that not only importin β but also energy and Ran are required [62] and studies of others in cell culture indicate that proteolytic cleavage of pUL36 is needed for uncoating [66] (Figure 2c). The subsequent step of genome passage into the pore also rests largely unknown. However, the mode of HSV capsid assembly favors a mechanism similar to bacteriophages, in which the genome is filled into preformed pre-capsids requiring energy. In fact, herpes viral DNA is packed to near crystalline densities in the capsid [67] and opening of the capsid then causes genome ejection (Figure 2d). This is driven by repulsion of the densely packed DNA through the pore, which was visualized in vitro by atomic force microscopy using capsids and nuclear envelopes from *Xenopus laevis* oocytes [68]. The last step of genome entry is assumed to be caused by transcribing RNA polymerases pulling out the viral DNA into the nucleus (Figure 2e). Such a mechanism was described for the bacteriophage T7 genome release [69] and the homology to HSV genome entry is supported by the observation that the immediate-early genes access the nucleus first [70]. In summary, HSV capsids show a kind of disrupted nuclear import reaction via transport receptors and additional direct Nup interaction, followed by a defined capsid opening at the cytosolic face of the NPC.

**Figure 3:**
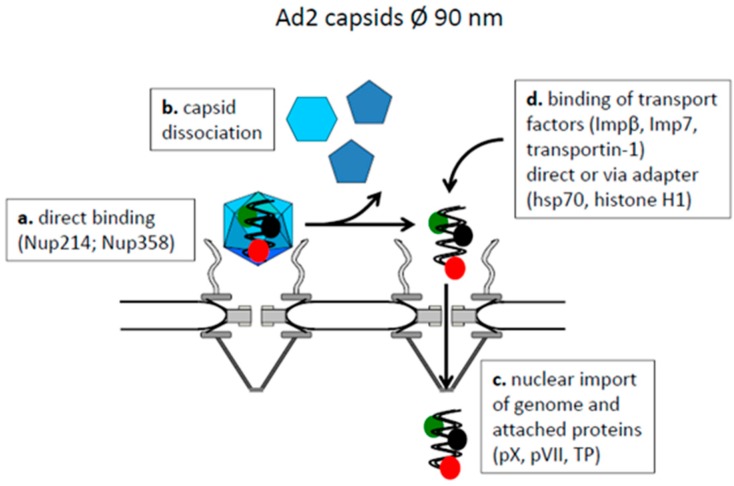
Model of capsid attachment to the NPC, capsid dissociation and nuclear genome transport of adenoviruses. Cytoplasm: top, karyoplasm: bottom. The capsids are shown as blue icosahedra, the genome as a double waved line in black. The genome-attached proteins are depicted as red, green and black spheres.

The non-enveloped Ads exhibit diameters of 90 nm and enter cells by clathrin-mediated endocytosis. They need acidification and proteolytic capsid modification for successful infection [71], which leads to removal of the viral fibers and part of the viral protein VI. Protein VI then mediates the release of the partially disassembled capsid into the cytosol and the partially dismantled capsid interacts directly with Nup214 [72,73] (Figure 3a). Using permeabilized cells, partially depleted for Nup358 or Nup214, Cassany et al. showed that Nup214 is required for capsid binding to the NPC but also for genome release and genome transport into the nucleus [74], while Nup358 was dispensable. These results are in conflict to data of Strunze et al. showing that Nup358 is involved in capsid disassembly. The authors further showed that genome liberation is driven by kinesin-1, a motor protein for anterograde cytoplasmic cargo movement [75]. As the authors also observed an increased permeability of the nuclear envelope upon Ad2 infection, they concluded that the kinesin-1 movement results in dissociation of Nups as Nup358 from the NPC. However, kinesin-1 movement raises forces of maximal 5.5 pN [76] but rupture of individual protein molecules interactions mostly need much higher forces (several ten or hundred pN; reviewed in [77]), so that the driving forces leading to genome release need confirmation.

During Ads genome release, the capsid fall apart to pentons and hexons, which stay cytoplasmic [78,79] (Figure 3b). This cytoplasmic localization is consistent with a transport receptor-independent capsid NPC-interaction, as an import receptor would mediate nuclear translocation. The released Ad genome remains attached to several viral proteins as protein VII, protein X and the terminal protein (TP) [80] (Figure 3c). While there is consensus that protein VII is essential for nuclear import of the genome, the molecular import pathway is less clear. As transport receptors importin β, importin-7 and transportin-1 were reported but the requirement of adapter proteins as heat shock protein (hsp)70 and histone H1 was described [72,81,82] (Figure 3d).

In summary, partially disassembled Ad capsids (i) interact directly with Nups; (ii) disassemble at the cytoplasmic face of the NPC into capsid subunits and (iii) the released viral genome becomes imported via genome-attached viral proteins by a not yet unequivocally identified classical nuclear import pathway using nuclear import receptors.

### 4.2. Capsids Smaller than the Maximal Transport Diameter of the NPC

A few capsids have diameters below the exclusion limit of the NPCs. *Circoviridae*, non-enveloped viruses with a single stranded DNA genome infecting pigs and birds, have diameters of 15 to 30 nm. They are composed of a single protein species (Cap protein) and exhibit a T=1 symmetry [83]. It was shown that both the Cap protein but also the viral replication protein Rep accumulate in the karyoplasm. Consistently, an NLS was identified on Cap but the requirement of the Cap NLS for nuclear import of the genome remains unknown.

**Figure 4:**
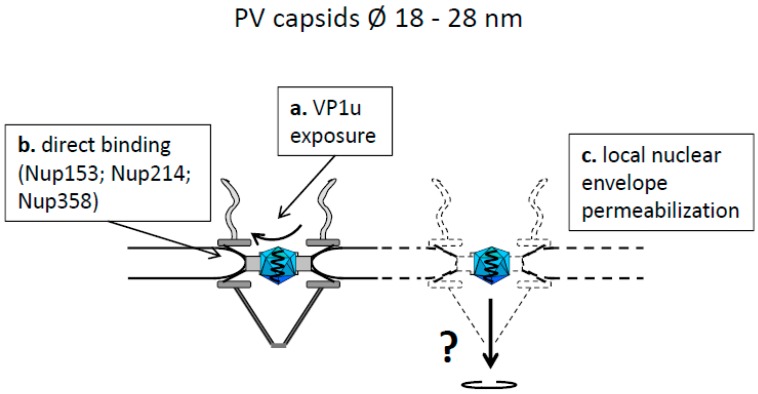
Model of parvoviral capsid attachment to the NPC and local dissociation of the nuclear envelope. Cytoplasm: top, karyoplasm: bottom. The capsids are shown as blue icosahedra, the genome as a single waved, black line.

*Parvoviridae* (PV), also non-enveloped viruses with a single stranded DNA genome and infecting a broad range of animals including human, exhibit capsid diameters of 18 to 28 nm (reviewed in [84]). They could thus pass the NPC without disintegration. This is seemingly supported by observations that parvoviral capsids accumulate in the nucleus shortly after infection, which is in agreement with the presence of an NLS on the large PV capsid protein VP1. However, the NLS which was found in different PV, localizes in the VP1u domain at the N-terminus, which is hidden in the virion (reviewed in [85]). Heat treatment externalizes this domain and it was proposed that such structural change also occurs upon endosomal entry and acidification. N-terminal VP1u exposure was also observed in vitro upon incubation of the parvovirus H1 (PV-H1) and adeno-associated virus 2 (AAV2) with isolated Nups [86] (Figure 4a) so that the mechanism of NLS exposure and its need for capsid interaction with NPCs stays unclear. An alternative NLS function is implied by parvoviral capsid assembly, which occurs within the nucleus requiring nuclear import of the capsid proteins VP1 and VP2. This transport is mediated by VP1, which forms a heterotrimer with two VP2 molecules, which are devoid of NLS [87].

Recent observation showed that the PV-H1, but also adeno-associated virus type 2 (AAV-2) attach directly to the Nup358, Nup214 and Nup153 (Figure 4b). It remains open if Nup153-binding reflects that the parvoviruses passed the nuclear pore in vivo as these assays were performed with disassembled NPCs. Considering the small capsid size, the direct Nup binding could result in an import receptor-independent transport similar to calnexin and calreticulin. However, PV-H1, canine parvovirus and different AAVs disintegrate the nuclear envelope locally [86,88], which was observed in several experimental systems and cell types including somatic cells and *Xenopus laevis* oocytes (Figure 4c). Local nuclear envelope degradation was not lethal to the cells but correlated to successful infection. It was shown to enhance nuclear entry of papillomaviruses, which are unable to trigger their nuclear entry during infection [8]. Parvoviruses thus combine characteristics of Ad, HSV-1, calnexin and calreticulin in terms of a direct interaction with Nups but they disintegrate the nuclear envelope instead of being imported into the nucleus by classical nuclear import pathways.

## 5. Organization of HBV Core Proteins and Capsids

HBV capsids are made up from a single protein species called core or capsid protein (Cp). Cp comprises a 140 amino acid-long structural domain and a 34–36 amino acid-long C- terminal domain (CTD), separated by a spacer of nine amino acids. The CTD harbors 16 arginine residues, organized in four clusters and five serine residues, which can be phosphorylated by a cellular protein kinase. The identity of the protein kinase is not unequivocally identified and the extent of Cp phosphorylation seems to be related to genome maturation inside the capsid. Furthermore the CTD comprises at least two overlapping bipartite NLS, which mediate nuclear import after fusion to bovine serum albumin (BSA) [89,90,91,92,93,94].

240 copies of Cp form a capsid with a T=4 symmetry even in vitro in the absence of other proteins. Upon Cp expression in *Escherichia coli* (coliC), a minor fraction exhibiting a T=3 symmetry was also described [95,96,97,98] but virion-derived capsids comprise the large form [99,100]. Assembly starts with rapid Cp dimerization, followed by a slow formation of hexamers. During the proceeding capsid assembly no further distinct intermediates could be identified [101,102]. Noteworthy, HBV capsids are not stable structures but are subject to permanent dissociation and re-association of Cp subunits, which is called capsid breathing [103].

Aside of their symmetry, HBV capsids can be further distinguished upon their nucleic acid content. (i) mature rcDNA- or double stranded linear (dsl) DNA-containing capsids (matC), which interact with the surface proteins and become secreted [104]. In our hands, these capsids are a minor fraction in transfected cells; (ii) capsids, containing the RNA pregenome and polymerase (Pol) (rnaC), which are also rare and which cannot be enveloped [4,104]; (iii) capsids containing all intermediates of genome replication from pregenome to rcDNA (immatC). As rnaC they do not become enveloped [4,104]. Of note is one exception: snow goose hepatitis B virions comprise a ssDNA genome [105]; (iv) empty capsids being a product of core protein over-expression (empC). These capsids can also be enveloped and secreted as empty virions [106,107].

As implied by their distinct capacity for envelopment, capsids not only differ in their enclosed nucleic acid but also in their structure. Within a resolution limit of 30 Å, coliC, which contain bacterial RNA, are identical to liver-expressed capsids [108] and more recent studies with 16 Å resolution supported that no gross structural changes are linked with genome maturation and envelopment [100]. However, better resolution with 10 Å showed that a hydrophobic pocket is present only on DNA-containing capsids [99].

Digestion with 40 nm gold particles-absorbed trypsin, which cleaves C-terminal arginine and lysine residues [109] was found to remove parts of the CTD [110] from capsids in which genome maturation has occurred. This was not observed when using coliC, which is consistent to image reconstructions from cryo-electron microscopy showing that the CTD is luminal [103]. In vitroCTD phosphorylation of these capsids (PcoliC), requiring dissociation, phosphorylation by protein kinase C (PKC) α/β and subsequent re-assembly, allowed trypsin digestion of the phosphorylated CTDs [110]. This indicates that the CTDs became exposed, which was recently confirmed by tryptic digests and cryo electron microscopy using a capsid mutant in which three serines of the CTD were replaced by the acidic aa glutamate mimicking phosphorylation [111]. The latter study also excludes that trypsin-cleavage of the PcoliC occurred due to the dissociation and re-association reactions.

The same tryptic digests were performed using matC, purified from cell culture supernatants of stably HBV transfected hepatoma cell line and with immatC purified from these cells. The results showed that proceeding of genome maturation was linked to increased CTD-exposure [110]. Digesting immatC from cells in which genome maturation was additionally inhibited, confirmed this finding and further showed that not the secretion process but genome maturation caused the increased CTD exposure. As demonstrated for PcoliC, trypsin digest removed all radioactively phosphorylated CTDs indicating similar or identical structural changes upon phosphorylation and genome maturation.

In summary, these data indicate that the CTDs on capsids have a dual topology—either inside the lumen or exposed to capsids exterior—which is driven by their affinity to the interior. In line with these findings Melegari et al. observed that Cp have a higher affinity to single stranded than to double stranded nucleic acids [112], explaining CTD exposure in mature capsids despite of their low phosphorylation.

## 6. Intracellular HBV Capsid Localization

Liver histology from HBV infected patients revealed that Cp and/or capsids are found in both cytosol and nucleus. Akiba et al. and Sharma et al. observed more frequent nuclear localization [113,114] while others observed a mainly cytoplasmic localization [115,116,117,118]. Clinically, the majority of studies associate cytoplasmic capsids with high hepatocellular injury [119,120,121,122] and a low level viremia [115]. Consistently, nuclear core dominance is associated with high viral load and minor hepatitis activity [120] but some patients show both core distributions.

Core from a patient with nuclear core stain was found to be assembled to capsids, and isopycnic CsCl ultracentrifugation showed that they were devoid of nucleic acids [123]. Consistently, electron microscopy of nuclei from core-transgenic mice exhibited a high concentration of nuclear capsids [124]. In these mice, cytosolic capsids were only observed in hepatocytes during cell division indicating that the nuclear envelope is impermeable for capsids. It was thus concluded that nuclear capsids are derived from nuclear import of unassembled Cp [124].

In HBV infected HepaRG cells but also in HBV-transfected hepatoma cell lines, core has been mainly detected in the cytoplasm but only occasionally in the nucleus [125,126]. Investigating the molecular mechanism determining capsid localization, Deroubaix et al. found that the relative expression of Cp and pol in the presence of the epsilon signal on the RNA pregenome determine Cp and capsid localization. The loss of interaction between CTD and Pol, which needs epsilon and Cp phosphorylation, caused nuclear capsid and Cp stain [126]. It remains however unknown which partner is rate-limiting in vivo as not only Cp but also Pol of HBV (and Pol the duck hepatitis B virus (DHBV)) is over-expressed [127,128].

## 7. Transport of HBV Capsids and Genome

Investigations on nuclear core transport need the specification of the protein structure, which is transported. Non-assembled Cp expose their CTD but phosphorylation may counteract importin α-binding to the NLSs similar to threonine 124 phosphorylation of the SV40TAg [38]. However, recent in vitro binding studies showed that unassembled core proteins exhibit a direct binding to importin β as the CTD also comprises an IBB [129]. As both, importin α-NLS and importin β-IBB interactions are based on electrostatic forces, the importin β-binding is stronger as more amino acids interact. Experimentally, the different affinities are in the µM range for NLS-importin α [130] and in the nM range for IBB-importin β [33]. Further problems in identification of core localization experiments arise from nuclear export of unassembled Cp due to a nuclear export signal found on HBV-but also on DHBV Cp [131,132].

Like Cp, empC also expose the CTD and also interact with importin β directly [129]. At high importin β concentrations, the capsids disassemble leading to the hypothesis that importin β is the key molecule for the removal of unassembled Cp and empC from the cytoplasm. Such removal could be important in infection as cytoplasmic Cp are degraded by proteasomes, which was observed upon addition of Cp-directed antivirals to HBV-expressing cells [133]. Entry of the proteolytic fragments into the major histocompatibility complex (MHC) class I pathway could then proceed via the endoplasmic reticulum (ER) and Golgi-bound TAP (transporter associated with antigen processing). The resulting exposure of core epitope on the surface of hepatocytes is known to be the main target of CD8+ T cells eliminating infected hepatocytes [134], which is in agreement to the inflammatory responses in patients exhibiting cytosolic core.

Consistent with the hidden CTDs, coliC cannot precipitate importin β neither directly nor indirectly via importin α from cytosolic lysates [89]. Consequently, they do not interact with the nuclear envelope in digitonin-permeabilized cells [89] or after microinjection into the cytoplasm of *Xenopus laevis* oocytes [29]. They thus do not show a significant interaction with outer parts of the NPCs as the capsids of adeno- herpes- and PV.

Adding PcoliC, phosphorylated to 0.5 phosphates per CTD, to permeabilized cells resulted in strong binding to the nuclear envelope and, more specifically, to the NPCs [89]. Surprisingly NPC-binding required not only importin β but also importin α. The need of importin α is supported by inhibition of the interaction by addition of an excess of NLS-peptides to which importin β does not bind [89]. Further, this finding is consistent with the observation that binding of importin β to the capsids needed importin α [89]. Together these findings imply that only the NLS-bearing part of the CTD is exposed but not in entire CTD, which is required for IBB-exposure. The alternative interpretation that phosphorylation interferes with direct importin β-binding but not with importin α-interaction appears unlikely due to the higher affinity of importin β for IBBs [130].

Early studies in cells showed that the NPC can import NLS-coated gold particles that are up to ~26 nm in diameter (including the protein coat; [135]), which was thought to be the threshold for karyophilic macromolecules crossing the NPC. Consequently, HBV capsids were assumed to become arrested on the cytoplasmic face of the NPC. It was thus in contradiction that PcoliC localize also in the nuclear basket after microinjection into the cytoplasm of *Xenopus laevis* oocytes [29]. Nuclear localization was not limited to the rare NPCs exhibiting a 10 fold symmetry suggesting that either the capsids had disassembled, followed by passage through the pore and subsequent re-assembly or that they had been squeezed, or that the maximal cargo size for macromolecules passing the NPC was seriously underestimated. Microinjection of NLS-coated gold particles confirmed that pore diameter was 39–40 nm, allowing passage of the capsids even after addition of an additional protein layer of importin α/β, which accounts for 1.9 nm [29]. These findings however raised the question why the capsids passed the NPC but failed to diffuse deeper into the karyoplasm.

Despite of several imaging data, showing that the structure of coliC and authentic capsids from patients or capsids from transfected cells are similar if not identical [95,112,136] it cannot be completely excluded that pol or heat shock proteins present in the capsids lumen modify capsids structure. This is in particular true as high resolution data were obtained by image reconstruction after cryo electron microscopy of thousands of capsids during which asymmetrical changes are merged out. Local structure changes e.g., at a site where Cp interact with pol would have been invisible, requiring confirmation of coliC-derived data by functional assays using authentic capsids.

Adding immatC to permeabilized cells showed that they bound to NPCs as PcoliC [110] and that they also entered the nuclear basket. MatC in contrast bound to the NPCs in a CTD- and importin α/β-dependent manner, entered the nuclear basket but were also found inside the karyoplasm [110].

Asking for the factor(s) retaining capsids in the nuclear basket, pull-down assays showed interaction with Nup153. Binding was ~200 times stronger than Nup153 interaction with importin β, which is a natural binding partner. Consistent with such strong interaction, partial silencing of Nup153 followed by nuclear import in permeabilized cells exhibited arrival of just a small proportion of PcoliC inside the nucleus [137]. However, the pull-down experiments with Nup153 revealed that also coliC efficiently bound Nup153. This indicates that importin α/β is just needed for capsid transport into the nuclear basket but that the CTD is not required for arresting the capsid.

The observation that NPCs can transport cargos with the size of HBV capsid into the nucleus does not exclude a more complex scenario: (i) capsids bind to the cytosolic face of the NPC where (ii) they disintegrate to Cp; (iii) followed by Cp translocation into the nuclear basket using nuclear import receptors; (iv) followed by reassembly to capsids after dissociation of the import factors on the nuclear side of the pore. Experiments in permeabilized cells and in *Xenopus laevis* oocytes in which UV-cross linked matC were used however showed that cross linking prevented arrival of matC in the nucleus although entering the nuclear basket [137]. These findings argued for a mandatory capsid disassembly before diffusing deeper into the karyoplasm.

In comparison to other viruses, HBV capsids thus have a more complex regulation in that there are different capsid forms, which differ in their genome maturation, Cp phosphorylation and structure. While exposure of the CTD is required for NPC interaction via nuclear transport receptors like it was shown for HSV-1 all capsids, direct interaction with Nup153 occurs after passage of the nuclear pore inside the nuclear basket. With regard to the latter interaction HBV capsids thus share also the characteristic of direct Nup binding as it was observed for Ad, HSV-1 and PV capsids.

## 8. Transport and Release of the Hepadnaviral Genome

### 8.1. Cytoplasmic Genome Release and Genome Transport by the Viral Polymerase

**Figure 5:**
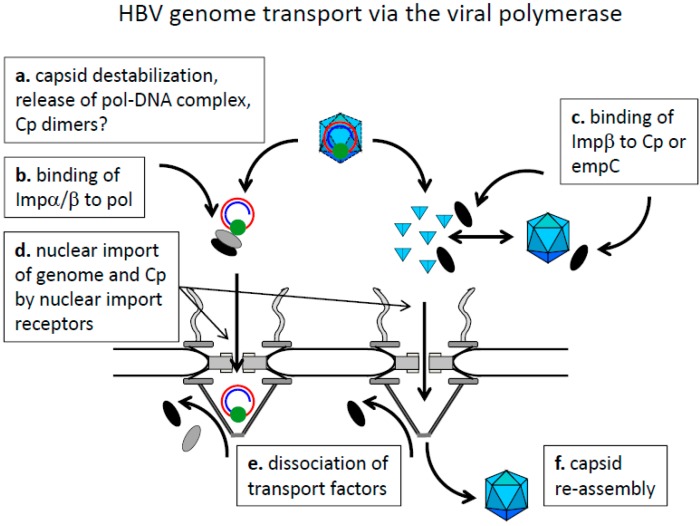
Hypothetical model of HBV genome transport mediated via the viral polymerase. Cytoplasm: top, karyoplasm: bottom. The capsids are shown as blue icosahedra, the genome as red circle (minus strand DNA) and an incomplete blue circle (plus strand DNA) with the covalently attached polymerase (green sphere). Importin β: black ellipse, importin α: grey ellipse. Core protein dimers are shown as light blue triangles. The model is based on a cytoplasmic capsid disassembly, which could also occur at the NPC. Capsid disassembly would result in core protein dimers, which in turn could re-assemble to empty capsids. These capsids would then be dissociated again upon importin β-binding. Import of the genome in complex with the viral polymerase is shown to occur via importin α/β as no IBB can be identified on the polymerase.

There are two possibilities of how hepadnaviral genomes could enter the nucleus, which are summarized in Figure 4 and Figure 5. The first model comprises nuclear genome entry driven by Pol, which is covalently attached to the genome (Figure 5). In fact exposure of Pol-genome complexes from the woodchuck hepatitis virus to isolated nuclei led to nuclear translocation of the genome [138]. However, isolation of the complex from the capsids required treatment with 4 M urea, denaturating Pol. This could lead to exposure of internal Pol domains, which are not exposed in vivo. Seemingly this assumption is supported by the findings of Cao et al., and Yao et al. showing that the Pol of HBV and DHBV are cytoplasmic [127,128]. Aside of fundamental differences to the woodchuck hepatitis virus Pol, the data however do not exclude that Pol undergo structural changes during encapsidation and genome maturation. Consequently, Pol structure released from the capsid could be different and in fact structural changes were reported upon epsilon binding [139,140]. Consistent with a Pol-mediated genome transport are findings of Guo et al., who expressed an envelope-negative HBV mutant in a hepatoma cell line [141]. The authors observed cytoplasmic DNase-sensitive rcDNA devoid of the viral polymerase which co-sedimented with capsids and which could be precipitated by anti-capsid antibodies. This indicates that capsids can open in the cytosol potentially allowing genome release and could result in Pol-mediated transport prior to pol degradation. This possibility remains however hypothetical as the majority of deproteinized genomes stayed capsid-bound favoring nuclear transport by the capsid. Further, it must be considered that the cytoplasm in these experiments was yielded by low speed centrifugation of a homogenate derived from douncing, which damages nuclei to some extent. As there was no contamination control of the cytosolic fraction with nuclear components the results require further confirmation.

There is thus no direct exclusion of a pol-mediated nuclear import of the HBV genome but the fact that HBV does not induce any innate immune response—nor counteract such a response [124,142]—indirectly argues against the release of a protein-rcDNA complex in the cytoplasm. Further support for a genome transport within the capsid comes from native fluorescence in situ hybridizations (native FISH). This technique allows detection of released HBV genomes only and showed no cytoplasmic but exclusively nuclear genomes after capsid-lipofection [143]. This technique induced high efficient cccDNA generation and subsequent virus replication indicating some similarity to the in vivo situation. However, the absence of cytosolic released genomes does not exclude capsid disassembly at the cytoplasmic face of the NPC followed by rapid translocation into the nucleus.

### 8.2. Nuclear Genome-Translocation in Intact Capsids and Nuclear Genome Release

Despite of the artificial setup, permeabilized cells not only allow the import of HBV capsids but native FISH revealed released intranuclear HBV genomes when matC were added [110]. This observation is in agreement with the disassembly of capsids to Cp dimers, which only occurred when the capsids got contact with the NPC [144]. When nuclear import was inhibited no capsid disintegration was observed with a threshold of detection which was estimated to be <5% of the subjected capsids.

**Figure 6:**
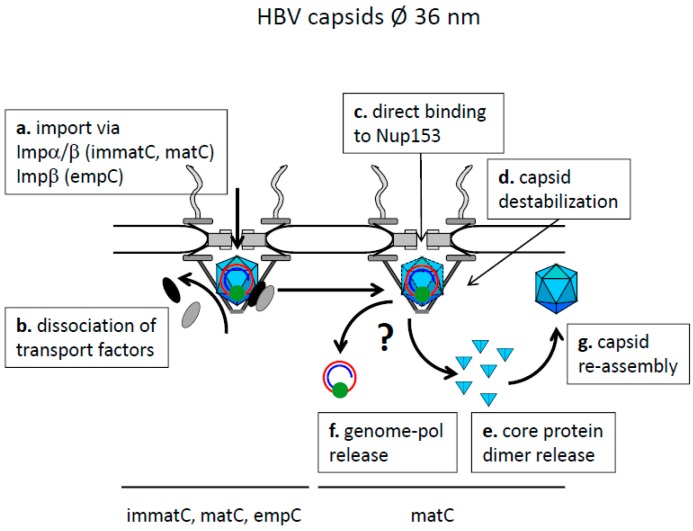
Model of HBV genome transport mediated in intact capsids. Cytoplasm: top, karyoplasm: bottom. The capsids are shown as blue icosahedra, the genome as red circle (minus strand DNA) and an incomplete blue circle (plus strand DNA) with the covalently attached polymerase (green sphere). Importin β: black ellipse, importin α: grey ellipse. Core protein dimers are shown as light blue triangles. The model is based on capsid disassembly in the nuclear basket after capsid Nup153-interaction. Disassembly is restricted to capsids with a mature genome by an unknown mechanism. It leads to release of the polymerase-genome complex and to nuclear core protein dimers, which re-assemble to empty capsids.

The latter studies, also summarized in Figure 6, showed that capsid transport and disassembly was combined with intranuclear re-assembly of the Cp dimers to genome-free capsids and no Cp degradation. The capsids were mostly filled with cellular RNA, which is in contrast to nuclear capsids isolated from human liver. As the liver capsids were likely derived from over-expressed capsids and not from genome import, a different Cp phosphorylation state could explain the difference. This hypothesis is in line with observations that these capsids contain one PKC molecule per capsid [145] and that PKC-phosphorylated core proteins do not encapsidate RNA upon in vitro assembly [146].

The electron microscopy data showing capsids in the nuclear basket suggest that this is the place where genome release occurs. Following this hypothesis, the disassembly of matC would result in 120 core protein dimers, which outnumber the 16 Nup153 molecules per NPC. Super numerous Cp dimers could then diffuse deeper into the karyoplasm and re-assemble to capsids. The limits of electron microscopy however do not allow to exclude that a part of the capsids leave the nuclear basket intact for disassembling shortly afterwards. Functional evidence against this hypothesis was shown by Rabe et al. [110], who observed that genome release, detected by native FISH, occurred even when Ran was absent, thus before a potential detachment of capsids could happen.

The observation that mature capsids disassemble preferentially can be explained with their lower stability as shown by Cui et al. [147] who used proteinase K resistance and DNase sensitivity as read-out of their in vitro assays. A potential molecular mechanism could be their low phosphorylation. In fact capsids, formed by the glutamate residue-mutant, were more stable to higher NaCl concentration [111]. The driving forces causing that the capsids with a mature genome disassemble in the nuclear basket remain however fully unknown.

Comparing the genome transport across the nuclear envelope of HBV capsids with that of other viruses thus shows obvious differences. Larger capsids as those of Ad and HSV have to disassemble on the cytoplasmic side of the NPC followed by classical nuclear import (Ad) or a mixture of injection and “pulling in” (HSV). Small capsids as that of HBV and PV can pass the NPC but they seem to stay bound by direct interaction with Nups. This raises the hypothesis whether the NPC is the general environment triggering genome release.
